# Human Urban Arboviruses Can Infect Wild Animals and Jump to Sylvatic Maintenance Cycles in South America

**DOI:** 10.3389/fcimb.2019.00259

**Published:** 2019-07-17

**Authors:** Luiz Tadeu Moraes Figueiredo

**Affiliations:** School of Medicine, University of São Paulo, São Paulo, Brazil

**Keywords:** yellow fever, arbovirus sylvatic cycle, arbovirus infecting non-human primates, arboviruses in brazil, reservoirs and vectors of arboviruses

## Abstract

The present study shows that the most prominent human arboviruses worldwide (dengue viruses 1, 2, 3, and 4, Chikungunya virus, and Zika virus) can infect wild animals and transfer from urban to sylvatic maintenance cycles in South America, as did the yellow fever virus (YFV) in the past. All these viruses are transmitted by the anthropophilic mosquito *Aedes aegypti* and cause epidemics throughout Brazil. The YFV is the oldest example of an urban arbovirus that became sylvatic in South America. Currently, the disease is a zoonosis of non-human primates that moves like a wave through the forests of the Brazilian countryside, traveling thousands of kilometers, killing many animals and eventually infecting man. However, since 2016, this zoonotic wave has reached the highly populated areas of Southeast Brazil, producing the largest human outbreak in the past 60 years. As with the YFV, sylvatic cycles may occur with dengue, Chikungunya, and Zika. In order to become sylvatic, arboviruses require an apparently unlikely conjunction of factors to unexpectedly take place. These arboviruses could start to infect sylvatic primates and be transmitted by *Haemagogus* mosquitoes that inhabit tree canopies. We mention here publications reporting evidence of sylvatic cycles of dengue, Chikungunya, and Zika virus in South America. Indeed, it is almost unfeasible to control these cycles of arboviruses since it is impossible to know where, when or why an arboviral spill-over would occur in wild animals. The sylvatic maintenance cycle could preclude the eradication of an arbovirus. Moreover, an arbovirus in a sylvatic cycle could re-emerge anytime, infecting humans and producing outbreaks. In case of the reemergence of an arbovirus, it is crucial to prevent the occurrence of an urban cycle as a spill-back from the sylvatic cycle.

Urban arboviruses such as dengue viruses 1, 2, 3, and 4 (DENV 1-4), Chikungunya virus (CHIKV), and Zika virus (ZIKV) can infect wild animals and transfer to sylvatic maintenance cycles in South America. This group of viruses also includes the yellow fever virus (YFV), which switched to a sylvatic maintenance cycle in the past (Figueiredo, 2007). All these seven arboviruses have urban maintenance cycles and spread throughout tropical areas of the world causing large epidemics of severe diseases with high significance in terms of morbidity and case fatalities. In around 10% of the infected individuals, YFV can cause severe hepatitis and hemorrhagic fever (Vasconcelos, 2003). All 4 DENV are causes of acute febrile illness and, in few cases, produce a severe disease due to capillary plasma leaking in the microcirculation that leads to shock and death (WHO, 2009). Patients infected with ZIKV virus generally present an acute febrile illness. However, 4 years ago, a severe congenital disease affecting the central nervous system was found in Brazilian children from mothers infected with ZIKV during pregnancy (França et al., 2016). CHIKV virus is also a causative agent of acute febrile illness, although many patients, especially women over 45 years of age, develop an arthropathy that chronifies with great impact on the quality of life (Nunes et al., 2015).

YFV, DENV 1-4, and ZIKV belong to the *Flavivirus* genera, and CHIKV to the *Alphavirus* genera, all of which are enveloped RNA viruses and constitute the most significant human arboviruses worldwide (Kuno et al., 1998; Forrester et al., 2012). All of them are originally from the Old World and were introduced into the Americas producing large outbreaks. YFV, ZIKV, and CHIKV were initially found in African primates, being transmitted by tree canopy-inhabiting mosquitoes. DENV 1-4 were originally viruses from primates of Southeast Asia that were also transmitted by these mosquitoes. All seven of these viruses, which retain high adaptative capacities, have currently begun to be transmitted by anthropophilic mosquitoes, such as *Aedes aegypti*, and to use man as a reservoir. Urban maintenance cycles have enabled their spreading in tropical areas of the world causing substantial epidemics (Hanley et al., 2013).

The forest proximity to urban environments in tropical countries has led to the entry of arboviruses from the wild into cities generating human disease, enhancing the risk to start urban maintenance cycles. In Brazil, Mayaro virus (MAYV, *Alphavirus*) and Oropouche virus (OROV*, Orthobunyavirus*) cause outbreaks in the Amazon, Central Plateau, and Pantanal regions. MAYV, an arbovirus of primates transmitted by *Haemagogus* mosquitoes that reside in treetops, stroke Manaus, a city with 2 million inhabitants, producing dozens of cases of acute febrile illness that had an initial diagnosis of dengue (Mourão et al., 2009). The same occurred with OROV, a virus whose sylvatic maintenance cycle involves sloths and primates, and whose urban cycle features the midge *Culicoides paraensis*. This virus has also caused outbreaks of acute febrile illness, including some cases of meningitis in Manaus city (Mourão et al., 2012). These data are merely to highlight that arboviruses can move back and forth from sylvatic to urban environments, and vice-versa (Figures 1,2). Thus, the aim of the present study is to stress that urban arboviruses in South America can move from cities to the wild, transferring from urban to sylvatic maintenance cycles.

**Figure 1:**
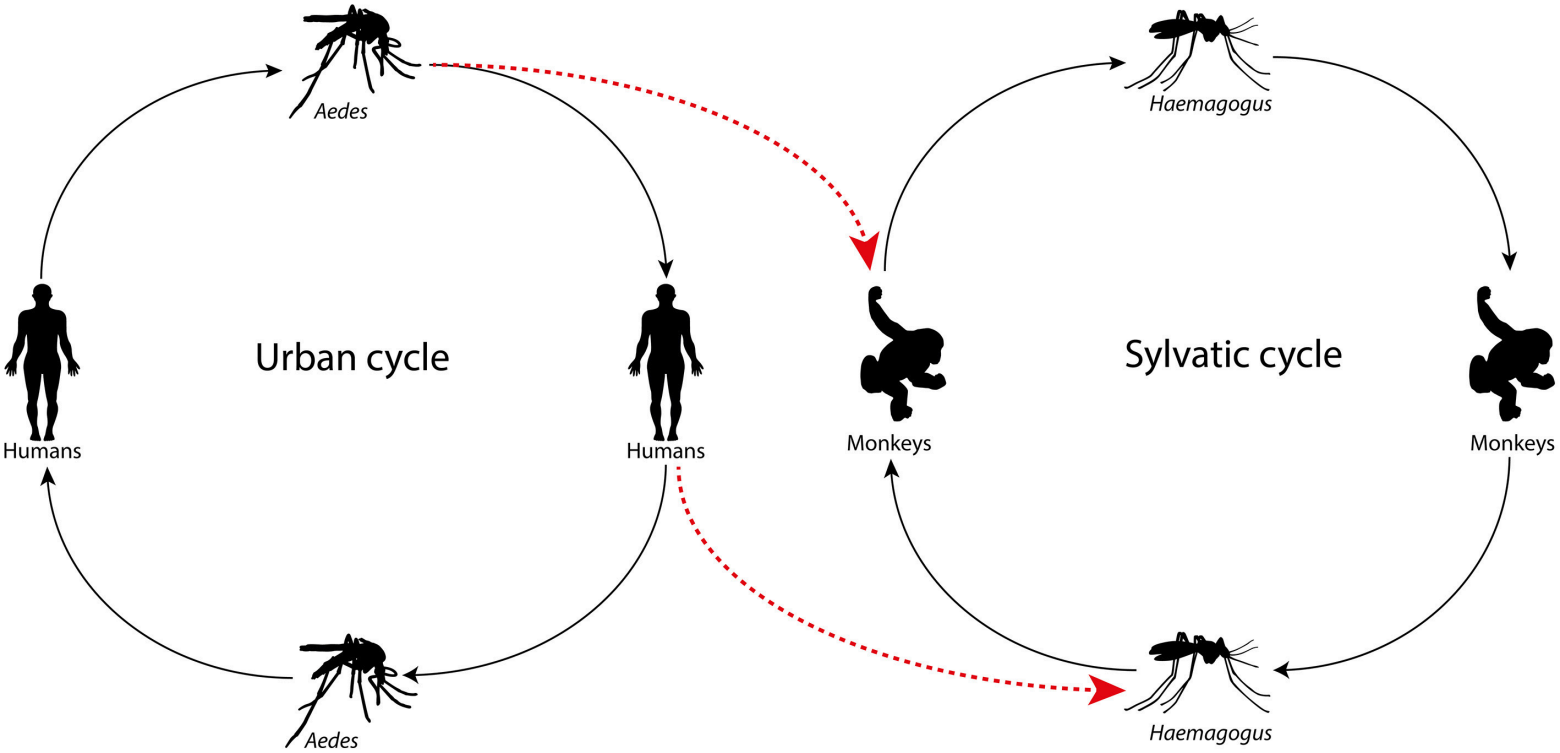
Arbovirus in the urban cycle jumping to the wild maintenance cycle due to the *Aedes aegypti* vector infecting non-human primates or viremic individuals infecting the wild mosquito.

**Figure 2:**
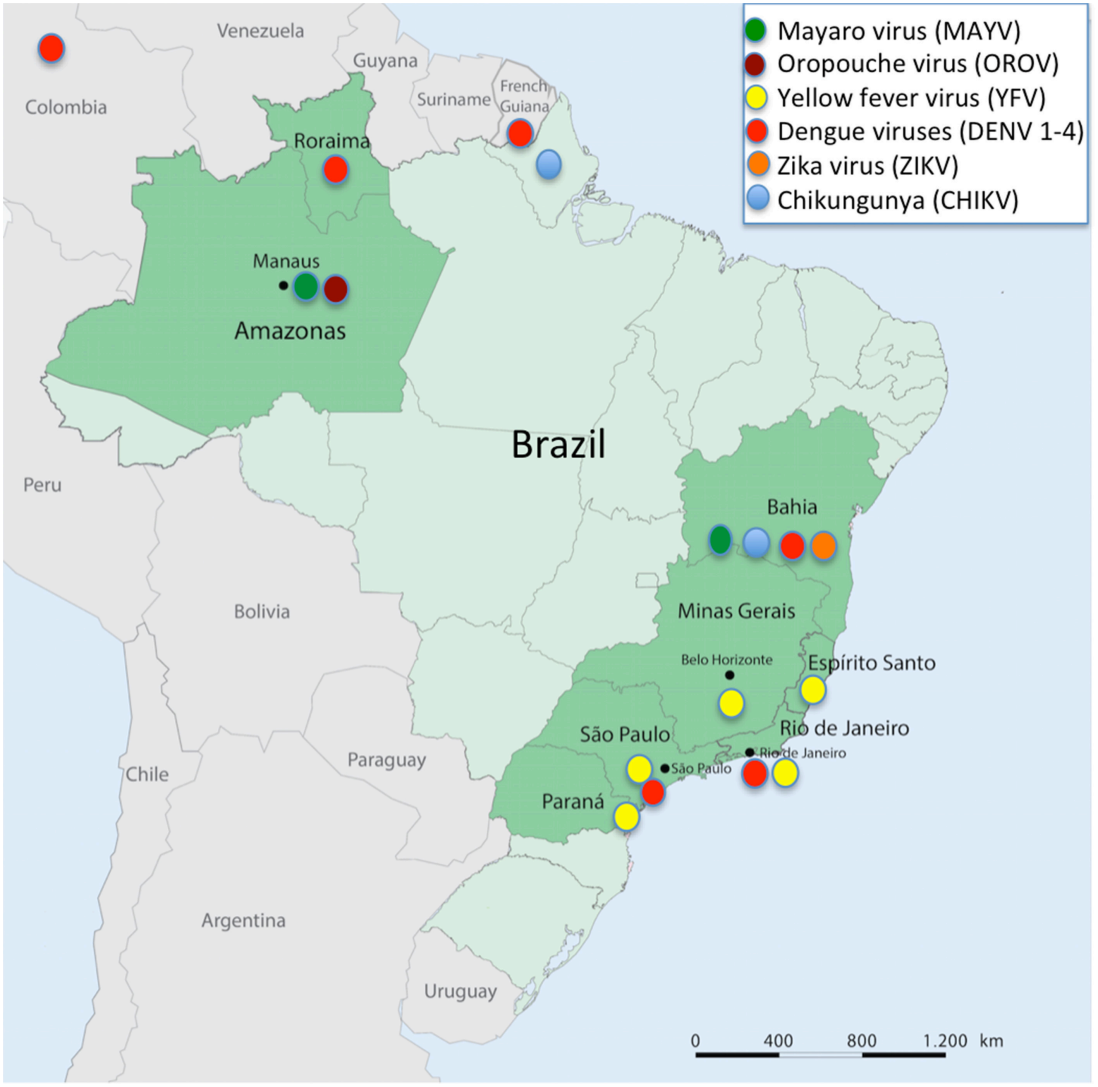
Partial map of South America showing Brazil, its States, and the neighboring countries mentioned in the text, also highlighting arbovirus infections reported at each mentioned site.

YFV was introduced into the Americas in the 16th century from Africa, probably as part of the slave trade in ships bringing sick viremic individuals, in addition to the vector *Aedes aegypti*. In the Brazilian Northeast, the virus produced large outbreaks in the 17th century, possibly transmitted by the same vector. At some moment between the 16th and 20th century, presumably as a consequence of more than one spill-over, YFV began to infect sylvatic primates and be transmitted by *Haemagogus* mosquitoes from tree canopies (Franco, 1976).

DENV has been the cause of a Brazilian catastrophe, with more than 10 million cases reported over the last 33 years. These viruses were introduced into the most populated areas of the country at different times, DENV-1 in 1986, DENV-2 in 1990, and DENV-3 in 2000, all of which were first reported in Rio de Janeiro. In contrast, DENV-4 genotype II was introduced into Roraima State in Northern Brazil in 2010, and, in the same year, produced large outbreaks throughout the country (Fonseca and Figueiredo, 2010). Currently, DENV 1, 2, 3, and 4 are all fostering alternated or simultaneous epidemics in different regions of Brazil. Regarding the sylvatic maintenance cycles, DENV 1-4 were reported to have infected neotropical forest mammals such as rodents, marsupials, and bats, in French Guyana (Thoisy et al., 2009), and in the Caribbean region of Colombia, and DENV-2 was reported infecting bats (*Carollia perspicillata* and *Phyllostomus discolor*) (Calderón et al., 2019). In Brazil, *Aedes albopictus*, a species of mosquito that frequently inhabits the backyards of human houses but easily spreads into rural, semi-rural, and wild environments, has been found infected with DENV-3 in São Paulo State. Meanwhile, in the State of Bahia, the sylvatic vector *Haemogogus leucocelaenus* was found to be infected with DENV-1 (Figueiredo et al., 2010). In another study carried out in the Atlantic Forest of Bahia, primates (*Leontopithecus chrysomelas* and *Sapajus xanthosternos*) were found with antibodies for DENV-1 and 2 and YFV, and sloths (*Bradypus torquatus*) had antibodies for DENV-3 (Catenacci et al., 2018). Therefore, despite not clearly shown, it is possible that sylvatic cycles of DENV occur in South America, although more studies are required to prove such a hypothesis.

CHIKV was introduced twice into Brazil. In 2013, the Asian lineage of CHIKV entered the North, whereas, the eastern, central, and southern African (ECSA) lineages were independently identified in 2014, and reported in Bahia State. A sequence of CHIKV outbreaks has stricken different regions of Brazil (Figueiredo, 2017). Studies have shown evidence that the virus could start a sylvatic maintenance cycle in the country. *Haemagogus leucocelaenus* and *Aedes terrens* have proven to become easily orally infected with CHIKV in the laboratory, suggesting high dissemination rates. *H*. *leucocelaenus* presents infectious viral particles in saliva and displays high rates of transmission 3 days after having an infectious blood meal. The competence of these sylvatic mosquitoes to transmit CHIKV was similar to those of several American *Aedes aegypti* (Lourenço-de-Oliveira and Failloux, 2017) (Figure 2). Moreover, in a serologic survey carried out in non-human primates of urban and peri-urban areas of Bahia State, 11 animals showed CHIKV neutralizing antibodies. However, two CHIKV-positive samples were also positive for MAYV, an arbovirus of primates that belongs to the same Semliki Forest antigenic complex of CHIKV that also causes cases of febrile illness with joint pain, producing small outbreaks or sporadic cases in North and Midwest Brazil (Moreira-Soto et al., 2018). The differential diagnosis between CHIKV and MAYV is crucial in Brazil (Figueiredo, 2015) to determine if sylvatic maintenance cycles of CHIKV could occur in South America. Further studies are required to prove such an assumption.

The Asian lineage of ZIKV was introduced into Brazil in 2013 and has affected thousands of individuals ever since. It is possible that the virus has the potential to establish a sylvatic transmission cycle in the Americas (Althouse et al., 2016). Many species of Brazilian primates and mosquitoes are potentially capable of transmitting ZIKV. In a serological survey carried out in non-human primates from Bahia State, 6 animals (*Atelidae, Callitrichidae*, and *Cebidae*) presented ZIKV neutralizing antibodies. However, 2 ZIKV-positive samples were also positive for DENV, suggesting cross-reactions among these flaviviruses (Moreira-Soto et al., 2018). In another study, mathematic modeling revealed a high probability of establishment of sylvatic ZIKV based on a broad range of parameters. The sylvatic transmission would depend on the sizes of the host and vector populations, in addition to the force of infection of ZIKV (Althouse et al., 2016). Studies on the competence of New World monkeys and other small mammals as ZIKV hosts and on that of *Sabethes* and *Haemagogus* as vectors are needed.

YFV is the oldest Brazilian example of an urban arbovirus that became sylvatic in South America. Today, yellow fever has shown to be a zoonosis of non-human primates that moves as a wave throughout the Amazon, Central Plateau, and Pantanal of Brazil, traveling thousands of kilometers and killing many animals, eventually leading to the infection of man in contact with the wild environment (Monath and Vasconcelos, 2015). However, since 2016, the zoonotic wave has reached the highly populated areas of the Brazilian Southeast, generating the largest epidemic in the past 60 years. Epizootics of yellow fever in primates have advanced through gallery forests, mostly along rivers of Southeast Brazil, reaching large cities such as Belo Horizonte (2 million inhabitants) in 2016–2017, as well as Rio de Janeiro (8 million inhabitants) and São Paulo (12 million inhabitants), both in 2017–2018. From January 2016 to June 2017, transmitted in a sylvatic cycle, yellow fever caused 3,140 cases with 410 deaths, mostly in the States of Minas Gerais, Espírito Santo, and Rio de Janeiro. In the second semester of 2017 and first semester of 2018, the epizootic moved southwestward, resulting in 1,266 cases and 415 deaths (Brazilian Ministry of Health, 2018). In 2018–2019, this ongoing epizootic of yellow fever bypassed the São Paulo metropolitan area and moved toward the coast, reaching the contiguous State of Parana in Southern Brazil (Brazilian Ministry of Health, 2019). The urban transmission of YFV by *Aedes aegypti* has not been reported during this outbreak (Figure 2).

Non-human American primates are highly susceptible to yellow fever, and many animals, namely howler monkeys (Alouatta), die as a consequence of this disease. In Minas Gerais State, it is estimated that 90% of the howler monkeys died from yellow fever in 2016–2017 (Lopes, 2017). The death of primates is a precocious detectable sign of yellow fever epizootics, and the surveillance of primate deaths in Brazil became an important tool to prevent cases of human yellow fever with the rapid vaccination of inhabitants in the target region. A computational analysis based on the surveillance of dead howler monkeys (more than 600 animals) and satellite photos enabled the mapping of ecological corridors and the progression rate of yellow fever among primates in São Paulo State in 2017–2018. Public health authorities state that such surveillance avoided the epizootic stemming from Minas Gerais State from reaching São Paulo City. The results guided a plan for previous immunization of the population with the YFV17DD vaccine before the arrival of the epizootic (Fioravanti, 2018).

During the yellow fever epizootic in southeastern Brazil, and in addition to taking care of thousands of human cases probably infected by sylvatic mosquitoes, public health authorities were significantly concerned regarding the spill-back of YFV returning to its urban cycle transmitted by *Aedes aegypti* in large cities, all of which are infested by this mosquito. Brazilian *Aedes aegypti* and *Aedes albopictus* are highly susceptible to both American and African YFV strains. Indeed, the reemergence of urban epidemics of YFV in South America could occur if the viruses were to be introduced either from a forest cycle or by a viremic patient from an endemic African region (Couto-Lima et al., 2017). Fortunately, Brazil benefits from an effective protector that avoids cases and also re-urbanization of yellow fever, the 17DD vaccine. This vaccine, containing live attenuated virus, is highly immunogenic and has been produced in Brazil since the 1940s (Frierson, 2010). The WHO recommends vaccine coverage of 80% or more of the population to impair urban outbreaks of yellow fever (Shearer et al., 2017). This sudden outbreak began in 2015 and stroke a community of the Brazilian Southeast that was poorly immunized to the virus. In order to fight against the issue, approximately 20,000,000 people were quickly vaccinated against yellow fever in 2017–2018.

Similar to YFV, sylvatic cycles may occur with DENV 1-4, CHIKV, and ZIKV. In order for this to happen, the maintenance cycle of an urban arbovirus requires an apparently rare conjunction of factors to occur: (i) an individual infected in the urban environment feeds a sylvatic mosquito (ex: *Haemagogus*) and infects this insect with the arbovirus or (ii) an urban *Aedes* feeds and transmits the arbovirus to a primate or another potential animal-reservoir; (iii) suitable amounts of the sylvatic vector-mosquito and the primate or other potential animal-reservoir inhabit the same ecologic niche and maintain close contact to promote and sustain the zoonotic cycle of the arbovirus (easily possible in South America, where wild primates and mosquitoes are abundant); or (iv) the animal-reservoir of the arbovirus maintains a suitable virus level in the blood to allow the infection of the vector mosquito. Moreover, eventually, a bridge-vector such as *Aedes albopictus*, which lives in urban areas but also spreads into rural, semi-rural, and forest areas, could carry the arbovirus to a sylvatic environment (Hanley et al., 2013).

As a form of protection concerning the emergence of new sylvatic cycles of urban arboviruses, antibody cross-neutralization, or immune responses, could abort infection with a virus of the same genus if concurrent infection of the primate occurred. Therefore, it is possible that the immunity of the primate against YFV could hinder infections by DENV and ZIKV and, thus, avoid their sylvatic cycles. Also, the competitive infection of arboviruses of the same genus in a vector could also exclude one of them producing the same effect (Moreira-Soto et al., 2018).

It is almost unfeasible to control sylvatic cycles of arboviruses, given it impossible to know where, when or why an arboviral spill-over would occur in wild animals. It inconceivable to tell if a spill-over infection will initiate the wild zoonotic cycle of an arbovirus. Moreover, it is considerably difficult to control infections in wild animal-reservoirs or sylvatic vector mosquitoes. On the other hand, an arbovirus in a jungle cycle could re-emerge at any moment, infecting human populations. Sylvatic transmission cycles for CHIKV and ZIKV involving non-human primates were reported in Africa, causing the cyclic reemergence of these viruses in humans. In case of arbovirus reemergence, it is crucial that patients be diagnosed and treated, and that an urban cycle of the arbovirus due to a spill-back from the sylvatic cycle be prevented (Couto-Lima et al., 2017).

Several actions could be taken to anticipate that potential sylvatic cycles of DENV-1-4, CHIKV, and ZIKV re-originate urban transmission by *Aedes aegypti*. One critical factor should be the maintenance of arbovirus surveillance in wild animals and vector mosquitoes. As for YFV, it would be essential to vaccinate more than 80% of the population against DENV 1-4, CHIKV, and ZIKV. However, since no vaccines are currently available for these viruses, their urgent development is required. It is also crucial to devise fast and reliable methods for the diagnosis of arbovirus infections in humans and animals. Arboviruses originated from wild cycles must be analyzed for mutations, changes regarding virulence or particular clinical patterns in infected patients. Ultimately, it is fundamental to develop antivirals and other drugs to treat patients. Recently, encouraging studies *in vitro* and in laboratory animals have shown that Sofosbuvir, a drug used to treat Hepatitis C, is also effective against YFV, DENV, CHIKV, and ZIKV (Bonotto et al., 2018; Freitas et al., 2019).

In short, the establishment of sylvatic maintenance cycles of DENV, CHIKV, and ZIKV is possible and could already be taking place in South America, promoting their re-emergence in human outbreaks and hampering their eradication. In order to confirm these cycles, studies with reservoirs and vectors in the sylvatic environment are required.

## Author Contributions

The author confirms being the sole contributor of this work and has approved it for publication.

### Conflict of Interest Statement

The author declares that the research was conducted in the absence of any commercial or financial relationships that could be construed as a potential conflict of interest.
